# Green Polymeric Nanocomposite (KCl/SiO_2_/Xanthan/*Origanum vulgare*) for Multi-Scale Interfacial Stabilization and Permeability Preservation in Carbonate Petroleum Reservoirs

**DOI:** 10.3390/polym18162035

**Published:** 2026-08-21

**Authors:** Yaser Ahmadi, Mehdi Havasbeigi, David A. Wood

**Affiliations:** 1 Chemical and Petroleum Engineering Department, Ilam University, Ilam P.O. Box 69315/516, Iran; 2 DWA Energy Limited, Lincoln LN5 9JP, UK

**Keywords:** polymeric nanocomposite, *Origanum vulgare*, interfacial tension, permeability preservation, atomic force microscopy, carbonate reservoirs, asphaltene deposition

## Abstract

In carbonate petroleum reservoirs, permeability impairment caused by asphaltene precipitation and deposition remains a major challenge that limits long-term productivity. This study introduces a green polymeric nanocomposite (KCl/SiO_2_/Xanthan/*Origanum vulgare*, NCs) designed to control interfacial dynamics and preserve flow capacity in carbonate formations. Using a multi-technique approach—interfacial tension (IFT) analysis, atomic force microscopy (AFM), and rock-core, fluid-flooding experiments at simulated subsurface conditions—the NCs’ abilities were evaluated in terms of their potential to modify properties at fluid–fluid and fluid–rock interfaces. The NCs increased the CO_2_–brine/oil IFT slope in certain pressure regions by up to 40.77%. These results indicate competitive adsorption that stabilizes interfaces. Adsorption isotherms confirmed a monolayer mechanism with a high capacity of 294.12 mg/g. AFM topographic mapping revealed order-of-magnitude changes in surface roughness (reductions in average roughness by ~75%, root-mean-square by ~83%, peak-to-valley by ~93%). These results directly link nanoscale smoothing to reduced capillary pinning. Core flooding tests demonstrated that NCs treatment decreased formation damage by up to 67.45% at 4000 psi, maintaining a high permeability ratio (k/k_i_ = 0.87) and preserving porosity (φ/φ_i_ = 0.887, representing 88.7% porosity retention). These results establish that the studied NCs coherently manipulate fluid physics in relation to molecular adsorption and macroscopic permeability. Consequently, these NCs offer a sustainable, high-performance strategy for flow assurance and formation damage control in geological and geothermal reservoirs.

## 1. Introduction

The destabilization of colloidal systems in subsurface reservoirs poses a critical challenge to fluid production and injection operations across geological and geothermal engineering applications. Asphaltenes, defined by their distinctive solubility behavior—insoluble in light aliphatic solvents like n-heptane but readily dissolving in aromatic solvents such as toluene—represent a primary cause of formation damage when they precipitate due to pressure decline, temperature drops, or compositional changes [1]. These perturbations upset the colloidal stability according to Derjaguin–Landau–Verwey–Overbeek (DLVO) relationships and modifications thereof [2]. This triggers nucleation, flocculation, and deposition that compromise wellbore integrity and impair flow capacity through pore throat blockage and surface fouling [3]. The adsorption of asphaltenes is governed by hetero-atoms (oxygen, nitrogen, sulfur). These atoms facilitate surface attachment through hydrogen bonding and π-π interactions. Moreover, solvent polarity, native resin concentration, and asphaltene molecular architecture are identified as critical controlling parameters [4].

Nanoparticles (1–100 nm) have shown considerable promise in petroleum engineering. Recent studies have also emphasized the role of surface modification in controlling interfacial interactions, with polymer and nanoparticle hybrid systems demonstrating enhanced performance in reservoir applications [5]. Their high surface-area-to-volume ratio enables wettability alteration, interfacial tension reduction, and improved hydrocarbon displacement efficiency [6,7,8]. Nanocomposites advance this further by integrating nanoparticles within polymeric matrices to create dual-mechanism systems. In such systems, nanoparticles adsorb asphaltenes, whereas polymeric components simultaneously provide steric repulsion and rheology modification [9]. Assessment methods used to reveal these relationships include CO_2_–crude oil interfacial tension (IFT) measurements, ultraviolet–visible (UV-Vis) spectroscopy, and adsorption isotherm analysis [10,11,12,13,14] have demonstrated nanoparticles’ efficacy. For example, magnetite nanoparticles are able to substantially inhibit asphaltene-onset pressure related to CO_2_ injection [15]. On the other hand, polymers have many applications in industry [16,17,18], and nanocomposites containing biopolymers, including xanthan gum, are particularly effective [19,20,21,22]. Manshad et al. [23] revealed that a KCl–SiO_2_–xanthan-gum NCs achieved 73.35% oil recovery but that study did not investigate asphaltene control. In reality, structure of permeability significantly influences fluid flow pathways and damage propagation in heterogeneous reservoirs [24]. Another important factor is pore-scale effects, which may contribute to the observed adsorption behavior in carbonate media [25], as demonstrated by advanced CT-based reactive transport modeling of porous carbonate dissolution [26].

Despite the mentioned recent advances in NCs applications, a unifying fluids-physics framework has yet to be fully developed. Such a framework is required to quantitatively connect NCs IFT phenomena with macroscopic-flow behavior in a range of subsurface conditions. The pivotal fluid dynamics aspect lies in the transition from asphaltene instability to pore-scale blockage, governed by (i) modification of the fluid–fluid interfacial stress tensor, (ii) alteration of colloidal forces between asphaltene aggregates and the rock surface, and (iii) evolution of surface topography that directly impacts hydrodynamic resistance and capillary trapping. This research addresses this critical gap by investigating a newly developed combination of KCl/SiO_2_/Xanthan/*Origanum vulgare* nanocomposites (NCs) on asphaltene deposition within carbonate pore networks. *Origanum vulgare* L., a commercially important herb of the Lamiaceae family, is recognized as a rich natural source of phenolic monoterpenes and their precursors—carvacrol, thymol, gamma-terpinene, and p-cymene—which exhibit high interfacial activity and potent antioxidant properties, making oregano extract an ideal natural reducing, capping, and functionally active agent for green nanocomposite synthesis [27,28].

The novelty of this work lies in its integrated multiscale fluid physics approach, which directly correlates nanoscale surface interactions and IFT modulation. These are quantified using AFM and pendant-drop tensiometer measurements. Moreover, the dynamics of multiphase flow and permeability preservation are observed in core-flooding experiments conducted under realistic pressure depletion scenarios. Building on our previously reported synthesis and characterization of KCl/SiO_2_/Xanthan/*Origanum vulgare* NCs [29], this study presents a new application domain focused on asphaltene-induced formation damage control. Quantitative links between adsorption thermodynamics, surface roughness evolution, and pore-scale fluid-flow dynamics governing permeability preservation in carbonate reservoirs were established. This represents a distinct scientific contribution beyond our prior work on methane foam stabilization [29]. It also introduces rigorous evaluation of permeability enhancement using nanocomposite inhibitors under realistic reservoir conditions. The data generated represents a significant advancement on the information provided by prior studies, which were confined to synthetic oils and idealized core samples.

The studied KCl/SiO_2_/Xanthan/*Origanum vulgare* NCs were recently investigated for their capabilities with regard to methane-foam stabilization, based on interfacial rheology and bubble-scale measurements to quantify morphological control [29]. The present work establishes a new application domain centered on adsorption thermodynamics, surface roughness evolution, and pore-scale, fluid-flow dynamics governing asphaltene-induced reservoir formation damage. This distinction is critical: foam stabilization relies on strengthening gas–liquid interfaces and retarding liquid drainage, while asphaltene inhibition demands adsorption of polar asphaltene moieties, modification of solid–liquid interfacial energetics, and nanoscale smoothing of depositional surfaces. Employing a multi-technique approach utilizing UV-Vis spectroscopy, AFM, CO_2_–oil IFT measurements, adsorption isotherm modeling, and natural depletion tests, this multifaceted methodology yields unprecedented insights into the KCl/SiO_2_/Xanthan/*Origanum vulgare* NCs impacts on asphaltene behavior under operationally relevant, sub-surface scenarios.

## 2. Materials and Methods

### 2.1. Materials

Crude oil and carbonate core samples were sourced from Iran’s Azar oilfield. This oil exhibits a viscosity of 11.69 cP at 86 °C, while analysis of a reservoir core revealed 15.1% porosity and 12.07 mD permeability. Carbonate substrate sheets (2 cm × 2 cm × 0.5 cm) were prepared from core plugs. The KCl/SiO_2_/Xanthan/*Origanum vulgare* nanocomposites (NCs) were prepared in our laboratory. The following reagents were employed for the synthesis: potassium chloride (KCl, ≥99%), sodium silicate (≥99%), sodium hydroxide (NaOH, ≥97%), n-heptane (≥99%), toluene (≥99.9%), and methanol (≥99.8%), all acquired from Merck KGaA (Darmstadt, Germany). Xanthan gum was supplied by Sigma-Aldrich (Burlington, VT, USA). All aqueous solutions were prepared using deionized water. *Origanum vulgare* L. leaves were collected locally and processed in-house for extract preparation.

### 2.2. Synthesis of Green Nanocomposite

The NCs were synthesized via a modified sol–gel method [29]. *Origanum vulgare* L. leaves (50 g) were washed, dried, ground, and macerated in 500 mL deionized water at 60 °C. The solution was maintained for four hours at constant stirring (500 rpm). The extract was filtered through Whatman No. 1 paper. Sodium silicate (5 g), serving as the silica precursor, was dissolved in 100 mL deionized water with stirring (400 rpm, 30 min). Oregano extract (50 mL) was added dropwise over 15 min. The solution was then subjected to sequential addition of KCl (2 g) and xanthan gum (1 g). The pH was adjusted to 10.5 using 0.1 M NaOH, and the suspension was stirred at 600 rpm and 70 °C for 6 h to promote hydrolysis and condensation of the silicate precursor, forming silica nanoparticles in situ. The product was then centrifuged (4000 rpm, 20 min), washed three times with deionized water, and dried at 60 °C for 24 h (yield: ~4.2 g). The detailed FTIR, XRD, SEM, and BET characterization of the synthesized NCs has been previously reported in our companion study [29]. Key characterization results are summarized here for completeness, while the present work focuses on the NCs’ application to asphaltene deposition control and permeability preservation.

### 2.3. Nanocomposite Characterization

FTIR spectroscopy was performed using a Thermo Nicolet NEXUS 870 spectrometer (Markham, ON, Canada), KBr pellets, 400–4000 cm^−1^, 4 cm^−1^ resolution. XRD analysis utilized a Siemens D5000 diffractometer (Munich, Germany), Cu Kα, λ = 1.5406 Å, 40 kV, 30 mA, over 5–80° 2θ. The Debye–Scherrer relationship was used to determine crystallite size. Scanning electron microscope (SEM) imaging was conducted on a Tescan MIRA3 FE-SEM (Brno, Czech Republic), 15 kV, after gold sputtering. EDX analysis was performed using an Oxford Instruments X-Max 80 detector (Abingdon, UK). Brunauer–Emmett–Teller (BET) surface area, pore volume, and pore diameter were determined by N_2_ adsorption–desorption. These measurements were derived at 77 K using a Micromeritics ASAP 2020 analyzer (Norcross, GA, USA), degassing at 120 °C for 12 h. Zeta potential was measured using a Malvern Zetasizer Nano ZS (Malvern, UK) at 25 °C (30 ppm NCs in deionized water, pH adjusted with 0.1 M HCl/NaOH). The NCs concentration of 30 ppm was selected based on a systematic optimization study (10–60 ppm range) reported in our previous work [29], where it consistently demonstrated optimal performance in terms of interfacial tension reduction, wettability alteration, and colloidal stability.

### 2.4. Atomic Force Microscopy Analysis

Surface morphology was characterized using a Bruker Dimension 3100 AFM in tapping mode (Bruker TESPA-V2 cantilevers, 42 N/m spring constant, 320 kHz resonant frequency, 0.5 Hz scan rate, 512 × 512 pixels). Carbonate core samples (2 cm × 2 cm × 0.5 cm) were submerged in 150 mL of asphaltene-toluene solution (25 mg/L) for 96 h at ambient conditions. Following solvent evaporation, the samples’ surfaces were analyzed to quantify asphaltene adsorption and topography alterations. Roughness parameters (R_a_, R_q_, R_t_) were calculated using NanoScope Analysis software (version 1.9) from five 10 × 10 μm scans per sample. For visualization purposes, representative cropped regions (approximately 1.2 × 1.2 μm) from the full 10 × 10 μm scans. However, the quantitative roughness parameters were calculated from the full scan areas.

### 2.5. Interfacial Tension Analysis

**Figure 1:**
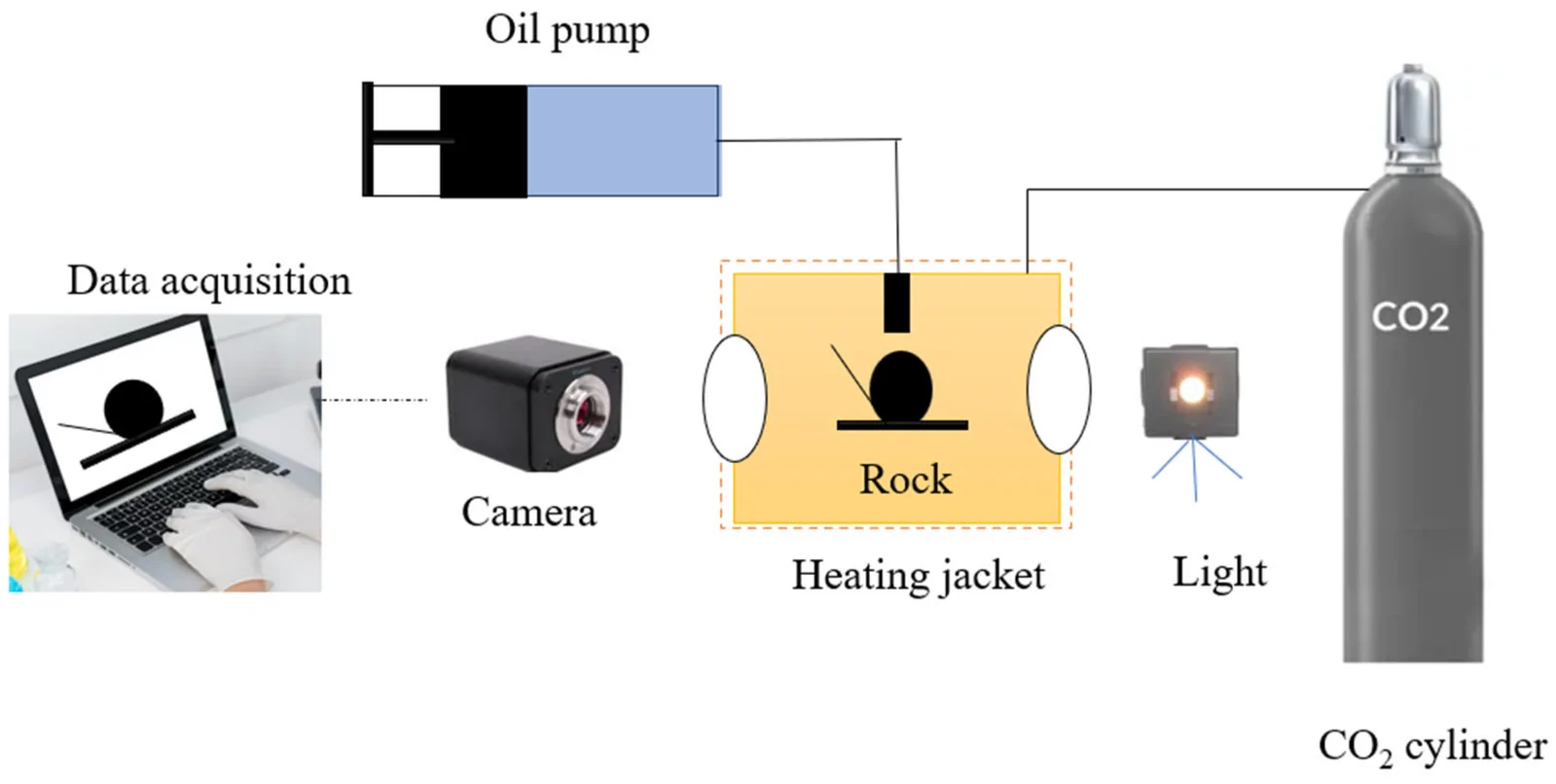
Schematic diagram of the high-pressure pendant-drop apparatus used for measuring interfacial tension between CO_2_ and crude oil.

IFT between CO_2_ and crude oil was determined by a high-pressure, pendant-drop tensiometer (Krüss DSA100, KRÜSS, Hamburg, Germany) at 86 °C and pressures up to 4000 psi, as shown in Figure 1. The equipment was cleansed using toluene and ethanol, and subsequently purged using nitrogen prior to analysis. CO_2_ injection was controlled using a Teledyne ISCO 260D syringe pump (Teledyne ISCO, Lincoln, NE, USA) (±0.01 mL/min). System pressure was monitored with Rosemount 3051 transducers (Emerson Electric, St. Louis, MO, USA) (±0.1% accuracy). Drop images were captured at 30 fps and analyzed using ADVANCE software (Version 1.11) with the axisymmetric-drop-shape analysis (ADSA) method. Each reported IFT value represents the mean of three repeated measurements. The densities of fluids in each analysis were determined with a NIST-certified hydrometers (±0.0001 g/cm^3^) with temperature control (±0.1 °C).

### 2.6. Batch Adsorption Studies

Asphaltenes were extracted from crude oil following the IP143 method. Stock asphaltene solution (1000 ppm in toluene) was diluted to initial concentrations ranging from 5 to 1000 ppm. NCs (30 ppm) were added to 50 mL of each asphaltene solution. The solution was then agitated at 200 rpm for 4 h at 25 °C in a shaking incubator. After centrifuging (3000 rpm, 30 min), the residual, supernatant, asphaltene concentration was measured with UV-Vis spectrophotometry (PerkinElmer Lambda 35, λ = 410 nm) using calibration curves (R^2^ = 0.999). Adsorption capacity (Q, mg/g) was then determined applying Equation (1):

Q = (C_0_ − C_e_) × V/m (1)

where C_0_ and C_e_ are initial and equilibrium concentrations (mg/L), V is solution volume (L), and m is nanoparticle mass (g).

### 2.7. Natural Depletion Experiments

**Figure 2:**
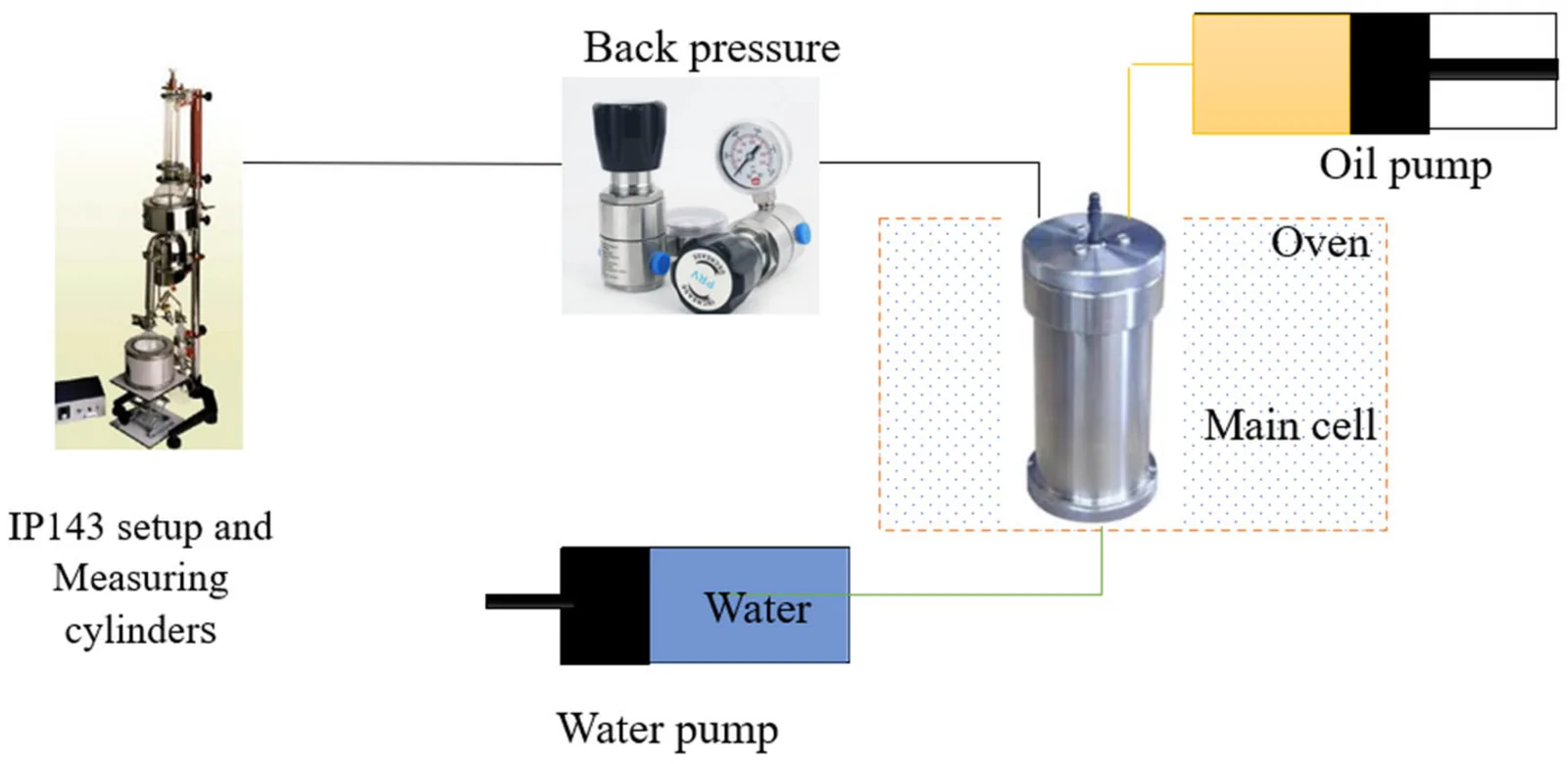
Schematic diagrams of the natural depletion apparatus setups used for asphaltene precipitation studies.

Natural depletion experiments were conducted using a high-pressure PVT cell (DBR Model, 5000 psi) equipped with a sapphire window, temperature jacket (±0.5 °C), and pressure transducer (Rosemount 3051, ±0.1% accuracy), as shown in Figure 2. Two sets of fluids were analyzed: (1) crude oil plus 30 ppm NCs, and (2) crude oil without NCs, functioning as a control system. The optimal NCs concentration of 30 ppm, as systematically determined through IFT, contact angle, and zeta potential measurements across a 10–60 ppm concentration range in our companion study [29], was employed in all subsequent experiments. This concentration consistently yielded the lowest IFT (10.54 mN/m), the most hydrophilic surface (contact angle of 39.58°), and the highest colloidal stability (zeta potential of −31.97 mV), confirming its selection as the scientifically optimized dosage. Both fluid sets (500 mL each) were saturated with CO_2_ at 86 °C and 4000 psi for 21 days. Depletion was performed in three stages (4000, 3700, and 3500 psi) at a rate of 50 psi/min using a QX-6000 pump (Chandler Engineering, Broken Arrow, OK, USA) (±0.0001 mL/min). At each stage, 10 mL samples were collected and filtered using 0.5 μm of stainless steel membranes. Asphaltene content was determined using the IP143 method: n-heptane addition (40:1 *v*/*v*), 24 h equilibration, filtration through Whatman Grade 40 paper, followed by toluene dissolution and methanol precipitation [30].

### 2.8. Core Flooding Experiments

**Figure 3:**
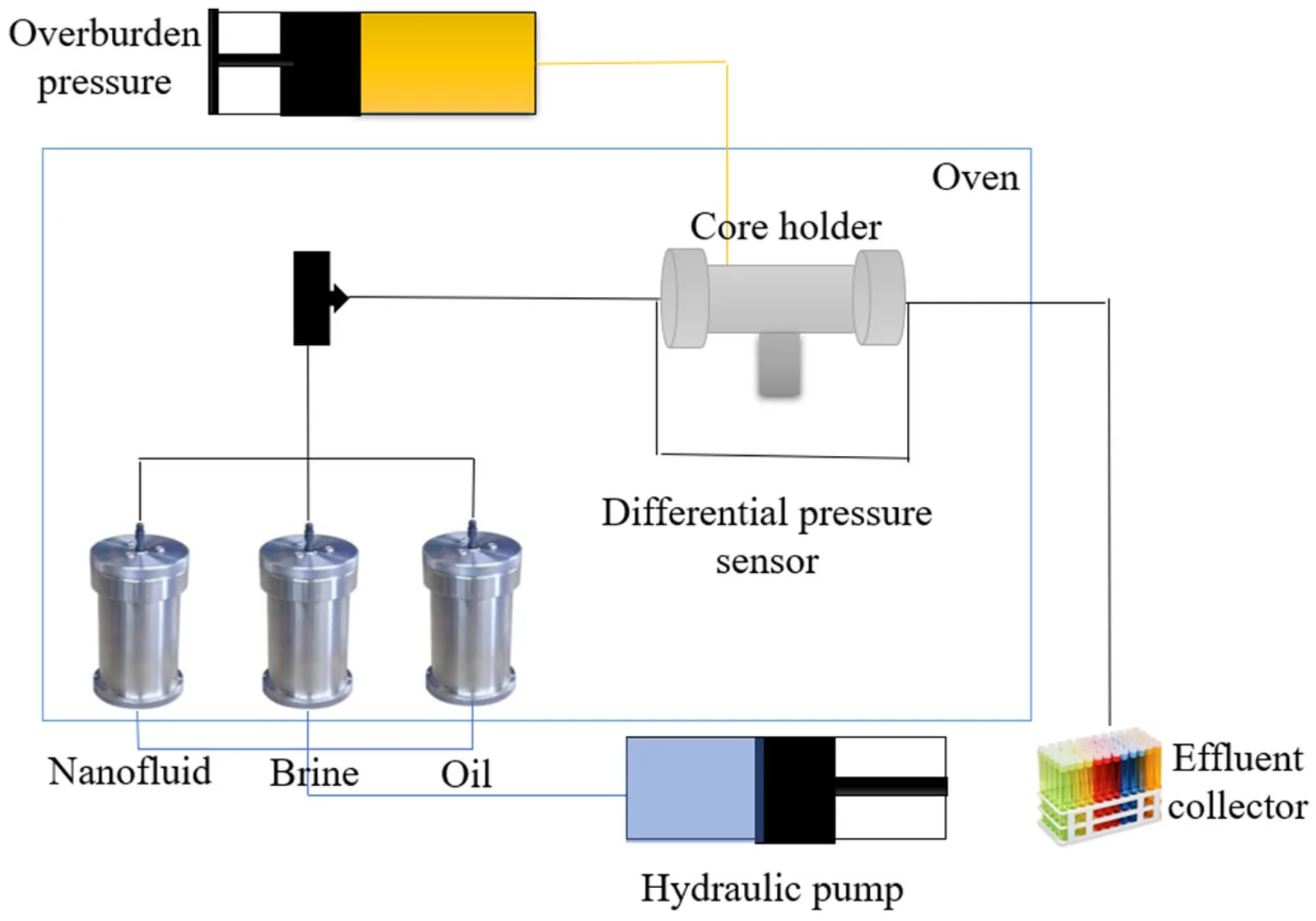
Core-flooding experimental system with Hassler-type core holder.

A Hassler core holder was employed for the core-flooding experiments conducted on the carbonate cores (length: 6 cm, diameter: 3.8 cm) from the Azar field (Figure 3). Cores were aged in crude oil for 21 days at 86 °C and 4000 psi pore pressure. Overburden (confining) pressure was maintained at 5000 psi throughout the experiments to ensure a positive effective confining stress of 1000 psi. Initial brine permeability was measured by injecting 4 wt.% NaCl at 0.1 mL/min under 2500 psi pore pressure. After establishing residual water saturation (S_wir_) through crude oil injection (1 PV/h), the initial effective permeability to oil (k_i_) was determined from the stabilized pressure drop at the start of oil flooding. The permeability ratio (k/k_i_) reported in Table 1 represents the ratio of effective permeability to oil at each pore volume to this initial oil effective permeability.

Two flooding scenarios were evaluated: (1) untreated crude oil samples (control), and (2) crude oil treated samples with 30 ppm NCs (equilibrated for 96 h at reservoir conditions). Both fluids were injected at 0.1 mL/min and 4000 psi back pressure. Effluent samples were collected at 0.5 mL intervals, and asphaltene content was analyzed using the IP143 method. The permeability ratio (k/k_i_) was calculated as the ratio of effective permeability at each pore volume (PV) injected to the initial permeability. Porosity reduction (φ/φ_i_) was estimated from the pressure drop data using Darcy’s law in conjunction with the Kozeny–Carman relationship [31], which relates permeability to porosity. Initial porosity (φ_i_ = 15.1%) was independently determined by helium porosimetry before flooding. The effective permeability at each pore volume was calculated from the pressure gradient, flow rate, and fluid viscosity. Porosity at each PV was then estimated assuming constant tortuosity and specific surface area. We acknowledge that this approach provides an estimated rather than directly measured porosity evolution; the reported φ/φ_i_ values should be interpreted as comparative indicators of porosity preservation rather than absolute measurements. All experiments were conducted under isothermal conditions (±0.5 °C) using PID-controlled heating jackets.

## 3. Results and Discussion

### 3.1. Characterization Results

**Figure 4:**
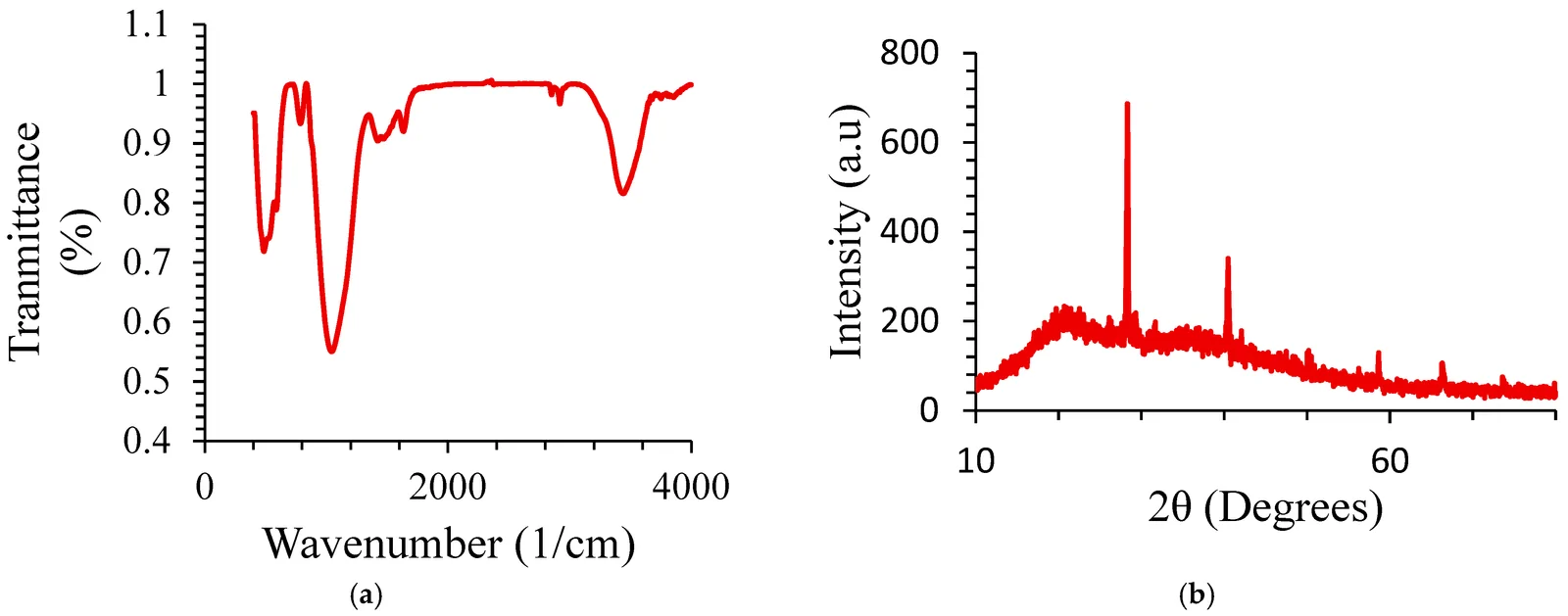
(**a**) FTIR spectrum displaying the characteristic absorption bands of the functional groups present in the synthesized nanocomposite; (**b**) XRD pattern of the nanocomposite showing crystalline phases and amorphous silica halo [29].

Fourier-transform infrared spectroscopy (FTIR) and X-ray diffraction (XRD) analysis were performed with Thermo Nicolet NEXUS 870 (KBr pellets, 400–4000 cm^−1^) and Siemens D5000 (Cu Kα, λ = 1.5406 Å, 40 kV, 30 mA, 5–80° 2θ) equipment, respectively. These analyses determined the functional groups and crystalline phases involved in each sample. The FTIR spectrum (Figure 4a) revealed characteristic bands at 3425 cm^−1^ (O-H/N-H stretching), 2917 cm^−1^ (C-H aliphatic), 1733 cm^−1^ (C=O carbonyl), 1618 cm^−1^ (C=C aromatic), 1058 cm^−1^ (Si-O-Si asymmetric stretching), and 894 cm^−1^ (Si-OH bending), confirming the organic–inorganic hybrid structure. The XRD pattern (Figure 4b) exhibited a broad amorphous silica halo (20–25° 2θ) superimposed with sharp crystalline peaks corresponding to quartz (SiO_2_) and sylvite (KCl), identified by ICDD PDF-2 matching (#96-901-2605 and #96-900-3130). Crystallite sizes ranged from 10.9 to 69.8 nm (Debye–Scherrer equation), with a volume-weighted mean of 49 nm [29].

**Figure 5:**
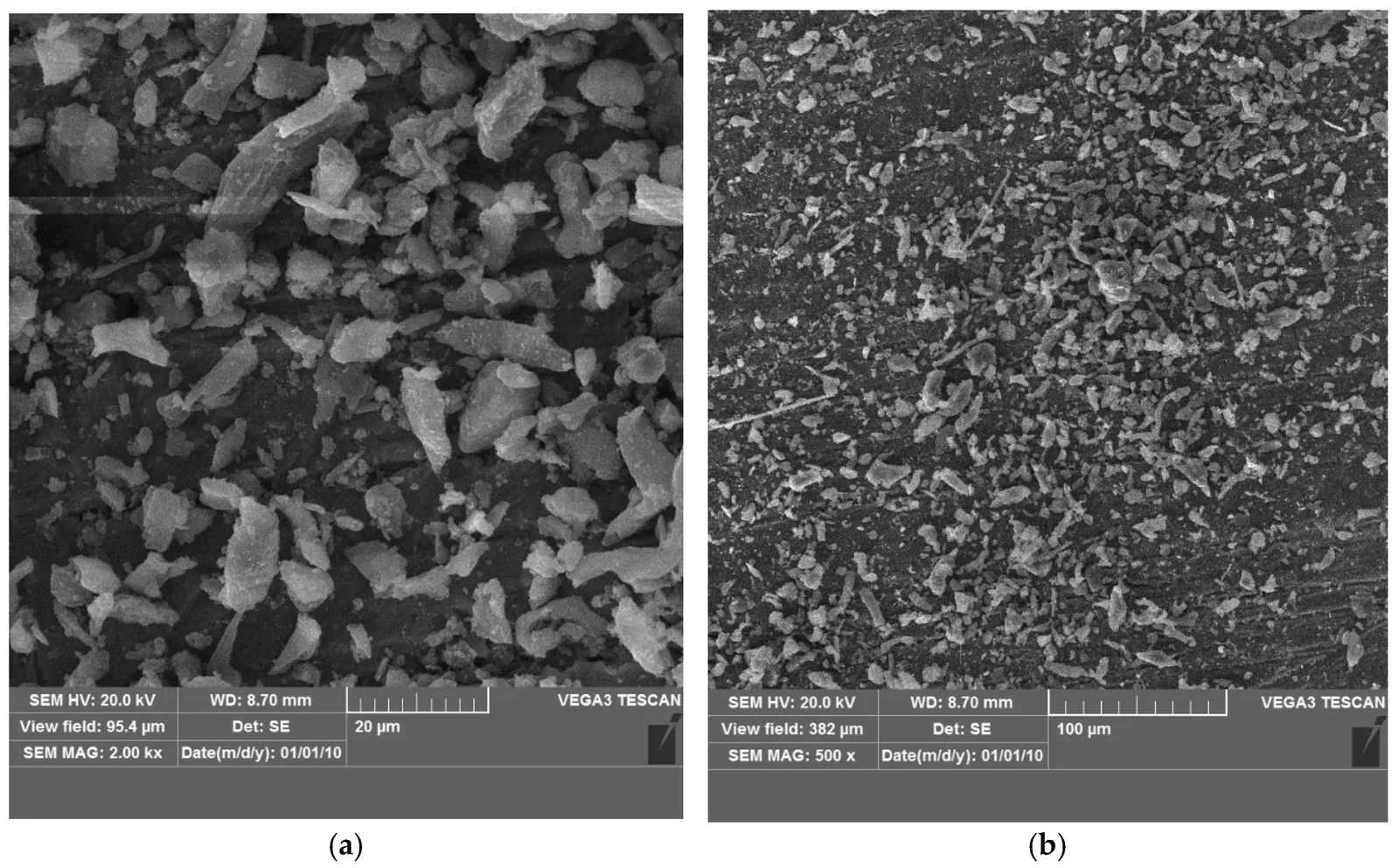
Scanning electron micrographs of the nanocomposite at different magnifications: (**a**) 20 µm scale and (**b**) 100 µm scale.

SEM Analysis: Morphological examination was conducted using a Tescan MIRA3 FE-SEM (15 kV, gold-coated samples). The micrographs (Figure 5) revealed homogeneous morphology with well-defined nanostructure. That morphological condition is interpreted as the results of the capping/stabilizing actions associated with the *Origanum vulgare* phytochemicals present [29].

Additional sample characterization was determined by BET surface area analysis (Micromeritics ASAP 2020, N_2_ adsorption–desorption at 77 K). Those measurements revealed 21.47 m^2^/g specific surface area, 0.0669 cm^3^/g total pore volume, and 12.47 nm average pore diameter, applying the Barrett–Joyner–Halenda (BJH) method, and classified the material as mesoporous. Energy-dispersive X-ray (EDX) analysis confirmed the elemental composition (C = 42.88 at.%, O = 38.48 at.%, Si = 5.22 at.%, Cl = 6.43 at.%, K = 5.70 at.%, and N =1.30 at.%). Zeta potential measurements (Malvern Zetasizer Nano ZS) characterized the sample’s surface charge properties. Further characterization details are reported elsewhere [29].

### 3.2. Adsorption Isotherms

**Figure 6:**
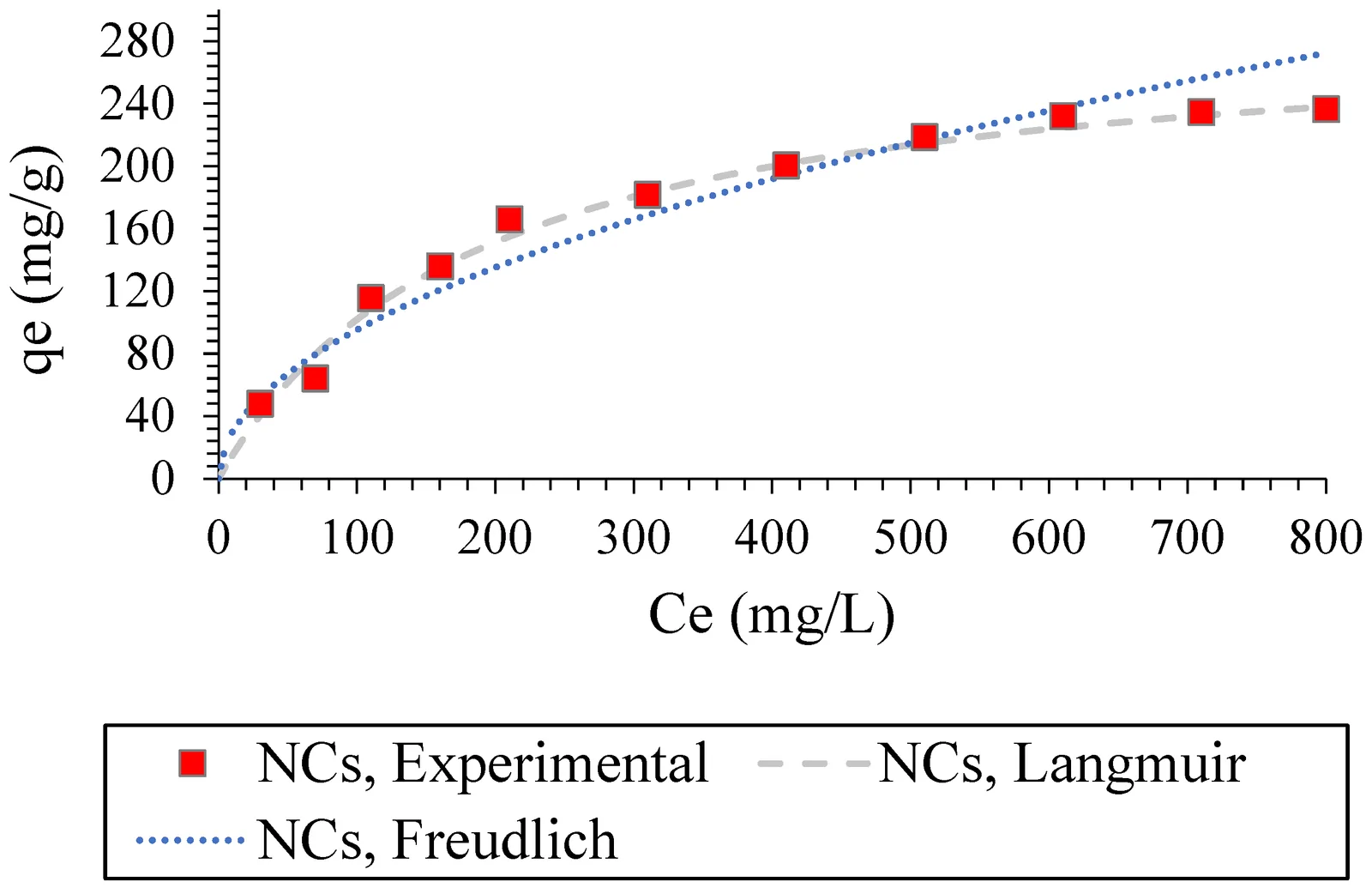
Adsorption isotherms of asphaltenes onto the nanocomposite surface, fitted with Langmuir and Freundlich models showing equilibrium adsorption capacity (Q_e_) versus equilibrium concentration (C_e_).

The experimental data was fitted with two isotherm models (Langmuir and Freundlich) to determine the asphaltene-adsorption capacity of NCs samples [32], as shown in Table A1. Figure 6 presents the adsorption isotherms for asphaltene concentrations ranging from 30–800 ppm, showing the relationship between equilibrium concentration (C_e_) and adsorbed amount (Q_e_).

**Figure A1:**
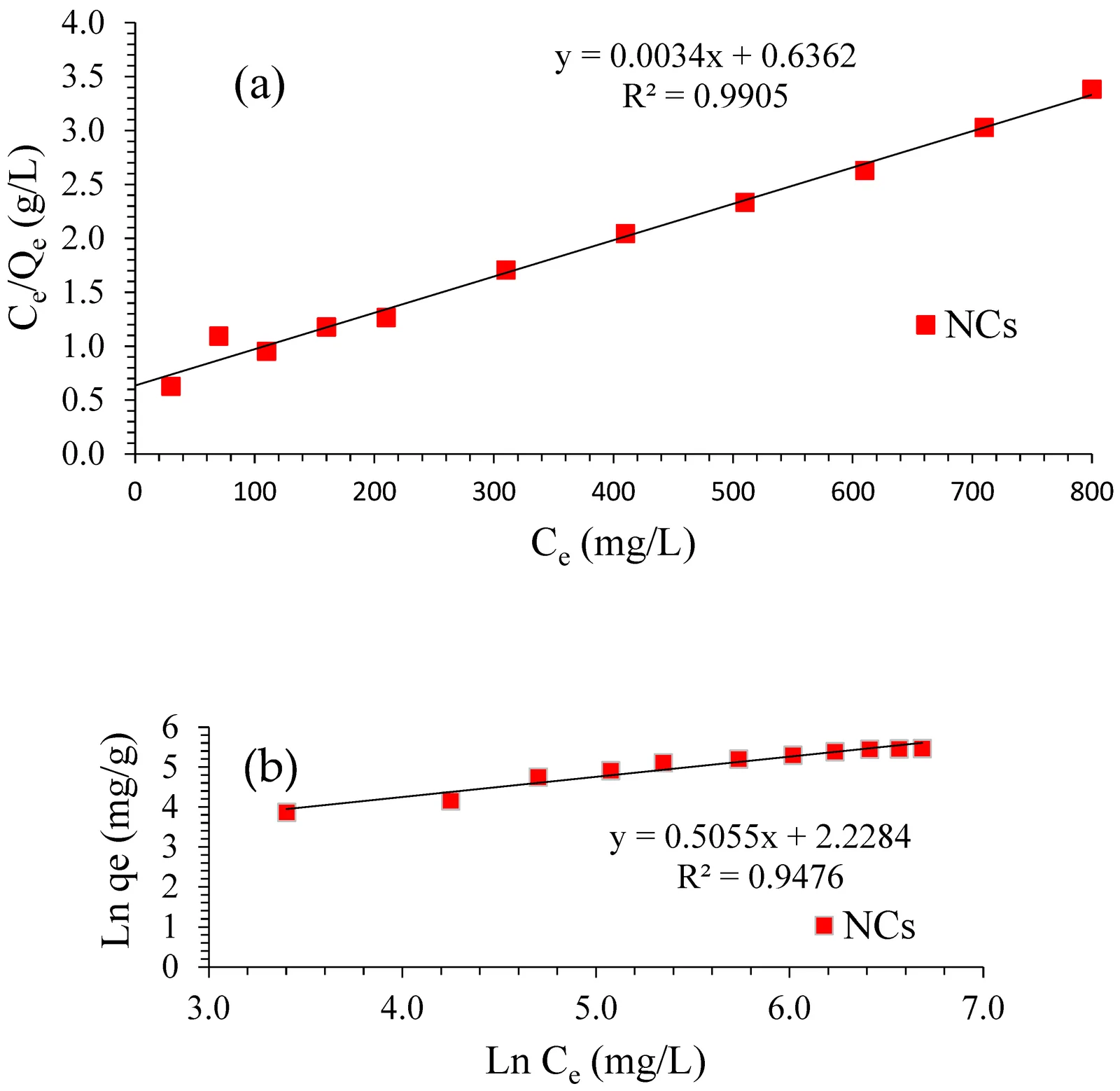
(**a**) Parameters of the Langmuir isotherm model for asphaltene adsorption onto NCs, (**b**) Freundlich isotherm determined for NCs.

The experimental adsorption data were fitted with Langmuir and Freundlich isotherm models. The Langmuir isotherm (R^2^ = 0.9905) exhibited a better fit compared to the Freundlich isotherm (R^2^ = 0.9476), suggesting that monolayer adsorption on homogeneous sites is the dominant mechanism under the investigated conditions (Figure A1a,b and Table A2) [32]. However, we acknowledge that isotherm model fitting alone cannot definitively confirm monolayer coverage. Other factors such as adsorbate–adsorbent interactions and surface heterogeneity may also influence the adsorption behavior. The calculated Langmuir affinity constant (K_L_ = 0.0053 L/mg) indicates moderate affinity between asphaltenes and the NC surface, consistent with reversible adsorption behavior. A more rigorous thermodynamic analysis would require temperature-dependent adsorption studies to determine enthalpy and entropy changes, which are beyond the scope of the present work.

To evaluate the contribution of individual components to the NCs’ adsorption performance, control experiments were conducted using silica alone, xanthan alone, and a silica/xanthan composite without oregano extract. All controls were tested under identical conditions (30 ppm equivalent concentration, 25 °C, 4 h equilibration). Table 1 reveals that silica alone and xanthan alone exhibited limited adsorption capacities of 45.2 and 28.7 mg/g, respectively. The silica/xanthan composite without oregano achieved a capacity of 112.3 mg/g, indicating that the polymer–nanoparticle combination enhances adsorption relative to individual components. The full NCs formulation demonstrated the highest capacity (294.1 mg/g), substantially exceeding all control systems. This enhanced performance is attributed to the combined effects of the silicate network (providing high surface area), xanthan gum (contributing steric stabilization and hydrophilic interactions), and oregano-derived phytochemicals (introducing additional hydrogen bonding and π-π interaction sites for asphaltene adsorption). These results indicate that each component contributes incrementally to the overall performance, with the oregano extract providing an enhancement beyond the silica/xanthan base system.

### 3.3. CO_2_/Oil-NCs Interfacial Tension

**Figure 7:**
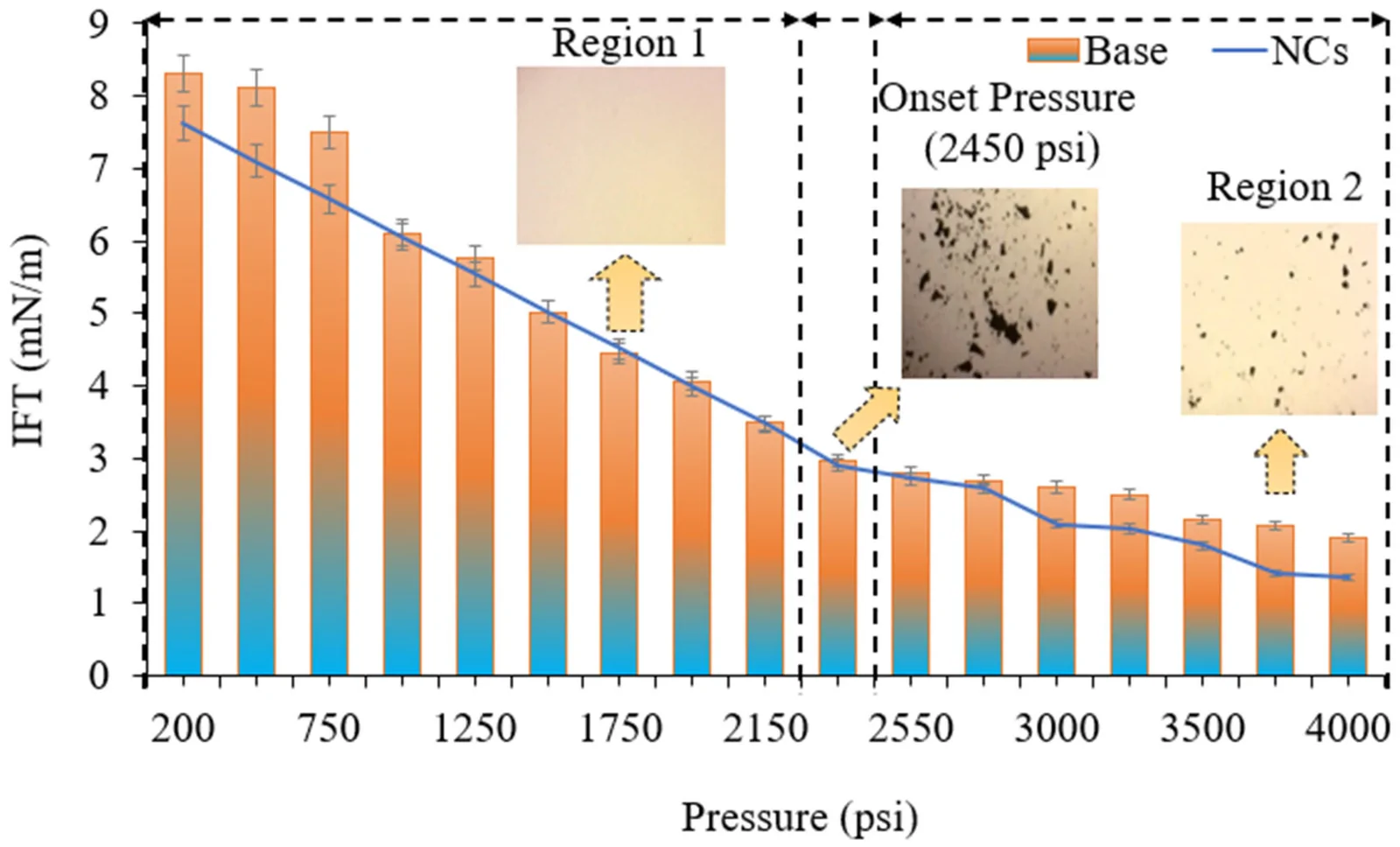
Interfacial tension behavior of CO_2_–crude oil systems as a function of pressure, comparing untreated crude oil (base) and nanocomposite-treated crude oil. The dashed vertical line at ~2450 psi marks the pressure transition where the IFT slope changes. High-pressure microscopy (HPM) insets show asphaltene particle morphology at representative pressures: Region 1 (below 2450 psi), where no asphaltene particles are observed, indicating stable asphaltene dispersion; onset region (~2450 psi), where asphaltene agglomeration begins; and Region 2 (above 2450 psi with NCs), where reduced aggregate formation is observed due to asphaltene adsorption onto NCs surfaces. The consistency between the IFT slope transition and HPM observations provides independent evidence that asphaltene destabilization contributes to the change in interfacial behavior, while the reduced aggregate formation above 2450 psi confirms the asphaltene adsorption capacity of the NCs.

Figure 7 displays the recorded variations in CO_2_–crude oil IFT in terms of pressure for NCs-treated and control (untreated) samples. Both systems exhibited slope transitions beyond 2450 psi, where IFT decline rate decreased. That decrease is interpreted to be a consequence of asphaltene precipitation. The presence of NCs modifies the IFT-pressure pattern to a substantial degree. This enhancement is achieved through an increased slope in the IFT-pressure relationship within the secondary pressure regime (above 2450 psi). The observed behavior is ascribed to the adsorption of asphaltene molecules onto the nanoparticle surfaces [16]. The ratio of IFT slopes increased from 26.92% to 45.45% (Table A3), indicating that NCs alter the interfacial energetics, likely through competitive adsorption of nanoparticles at the CO_2_–oil interface. This reduces the interfacial concentration of surface-active asphaltenes and delays critical aggregation pressure. This shift arises from competitive adsorption of nanoparticles at the CO_2_–oil interface, reducing interfacial concentration of surface-active asphaltenes and delaying critical aggregation pressure. Complementary UV-Vis analysis verified sustained asphaltene adsorption on NCs over 96 h. These observations align with those of Kazemzadeh et al. [15], who similarly reported nanoparticle-mediated IFT modification during CO_2_ flooding. Furthermore, recent digital rock studies have revealed the transport mechanisms of CO_2_/CH_4_ mixtures in carbonate systems [33], providing additional context for understanding the role of interfacial phenomena in CO_2_–oil interactions.

To provide independent evidence for the interpretation of this pressure transition, complementary high-pressure microscopy (HPM) analysis was conducted on pressurized oil samples during controlled depressurization in a sapphire high-pressure visual cell equipped with a microscope and camera system. HPM is a widely established technique for directly visualizing asphaltene particle formation and growth under reservoir conditions. It enables the determination of asphaltene onset pressure (AOP) through visual observation of particle count and size. The HPM observations (Figure 7 insets) revealed a clear pressure-dependent behavior. At pressures below approximately 2450 psi (Region 1), no asphaltene particles were observed, indicating stable asphaltene dispersion in the crude oil. At the onset pressure around 2450 psi, asphaltene agglomeration became detectable. Interestingly, in the presence of NCs at pressures above 2450 psi (Region 2), significantly reduced aggregate formation was observed, indicating that the NCs effectively adsorb asphaltenes onto their surfaces, preventing agglomeration. This pressure threshold (~2450 psi) is consistent with the IFT slope transition, confirming that asphaltene destabilization and subsequent adsorption onto NCs contribute to the observed changes in interfacial behavior. The presence of NCs modifies the IFT-pressure pattern to a substantial degree. This enhancement is achieved through an increased slope in the IFT-pressure relationship within the secondary pressure regime (above 2450 psi).

### 3.4. Natural Depletion Evaluations

**Figure 8:**
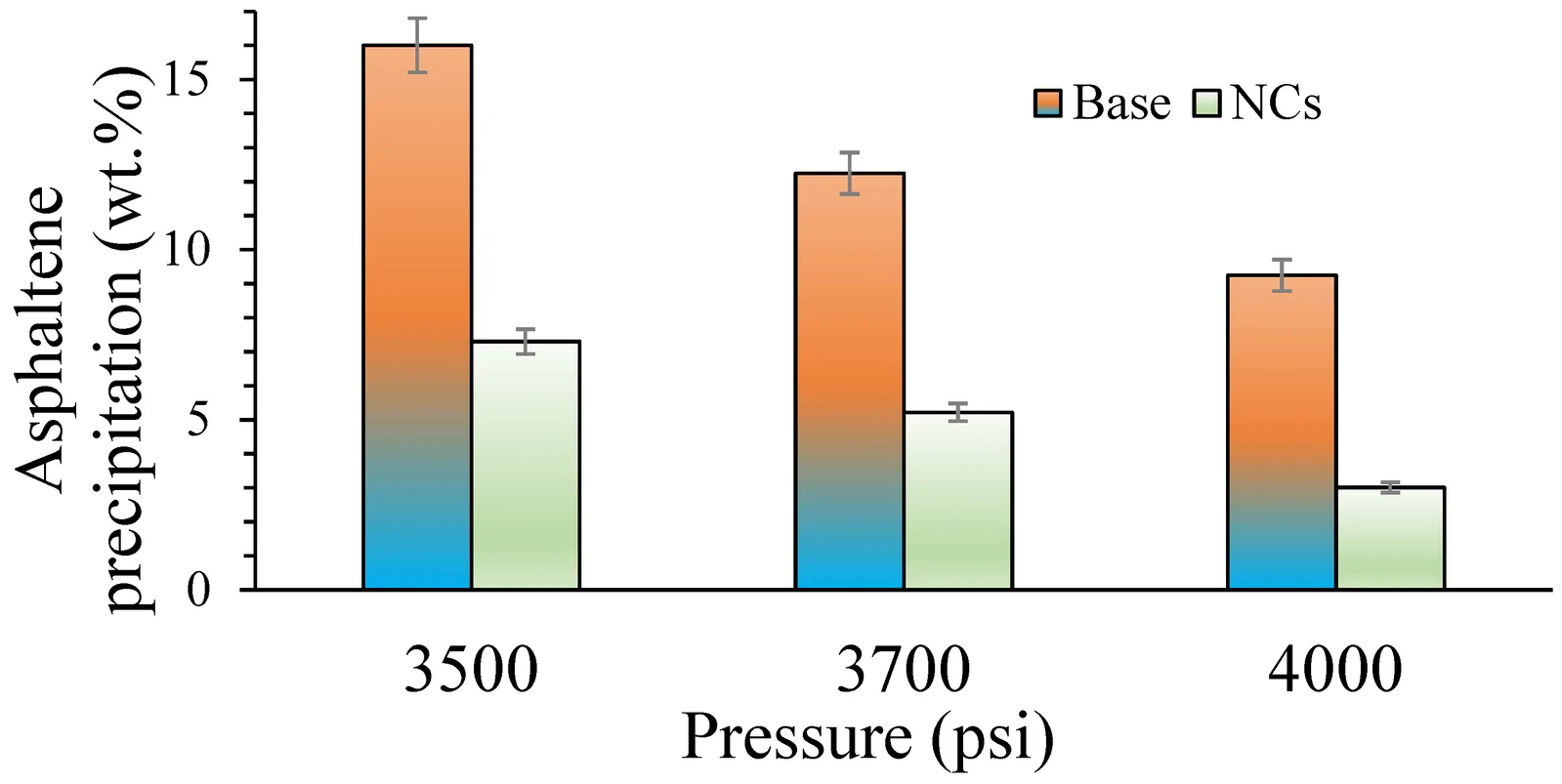
Asphaltene precipitation levels measured at three distinct pressure stages for the control (untreated) and nanocomposite-treated systems.

Systematic pressure reduction protocol evaluated three critical pressure stages (4000, 3700, and 3500 psi) to characterize precipitation behavior during progressive depletion at 30 ppm NCs concentration (Figure 8).

All systems exhibited increased asphaltene precipitation with decreasing pressure, consistent with conventional depletion behavior. However, NCs-treated systems displayed markedly reduced precipitation rates at every pressure point compared to untreated controls. At 3500 psi, NCs treatment reduced precipitation from 16.01 wt.% to 7.30 wt.%—a 54.40% reduction—while at 4000 psi, precipitation decreased from 9.25 wt.% to 3.01 wt.% (67.45% reduction). Sustained efficacy over 96 h demonstrates consistent performance without diminished returns, suggesting nanoparticles offer viable solutions throughout the production lifecycle.

### 3.5. Atomic Force Microscopy

Topographic analysis determined by AFM (Table 2) revealed significant differences in surface morphology between NCs-treated, carbonate core sheets and control (untreated) sheets following ninety-six hours of exposure to asphaltene solutions (25 mg). The base condition exhibited considerable roughness: R_a_ = 72.44 ± 1.18 nm, R_q_ = 67.65 ± 1.65 nm, and R_t_ = 246.25 ± 2.09 nm. In striking contrast, NCs-treated samples displayed order-of-magnitude reductions: R_a_ = 18.02 ± 0.18 nm (75.12% decrease), R_q_ = 11.12 ± 0.37 nm (83.56% decrease), and R_t_ = 16.22 ± 1.30 nm (93.41% decrease). This nanoscale smoothing minimizes capillary-pinning forces. It appears to achieve this by reducing hydrodynamic resistance in pore-throat locations within the samples, an interpretation that directly explains the observed permeability retention. The uniform distribution of results is consistent with the suppression of diffusion-limited aggregation kinetics, a phenomenon typically associated with rough asphaltene films. These findings confirm NCs promote homogeneous asphaltene deposition via surface energy modification, effectively preventing localized peak formation.

The AFM measurements were performed on carbonate coupons (2 cm × 2 cm × 0.5 cm) prepared from the same Azar oilfield reservoir core material as the core-flood specimens (6 cm × 3.8 cm). While these are separate specimens, they share identical mineralogy, pore geometry characteristics, and asphaltene exposure conditions (25 mg/L, 96 h), ensuring the adsorption behavior observed on coupons is representative of pore-wall adsorption within the flooded cores.

The observed order-of-magnitude roughness reduction (R_a_: 75.12%, R_q_: 83.56%, R_t_: 93.41%) is mechanistically linked to permeability preservation through well-established physical relationships. Independent studies have demonstrated that surface roughness causes interface pinning and capillary barriers in porous media [34]. Indeed, quantitative relationships show that a 0.1-unit increase in relative roughness elevates capillary pressure by approximately 20% [35], while a 0.025-unit increase reduces relative permeability by 10% for the wet phase and 18% for the non-wet phase [35]. Consequently, the roughness reduction achieved by NCs treatment directly translates to reduced capillary pinning forces and enhanced relative permeability. The relationship between interface roughness and permeability has been further validated in recent multi-scale studies [36], which demonstrate that roughness amplitude directly influences crack initiation and fluid flow pathways.

### 3.6. Multiphase-Flow Conditions in Porous Reservoir Samples

Core-flooding tests quantitatively verified the NCs’ performance in suppressing asphaltene-induced formation damage (Table 3). The differential pressure (ΔP) of the control (untreated) samples increased rapidly with injected pore volume. This was a consequence of severe pore-throat plugging and rapid permeability-ratio decline (k/k_i_). Conversely, in the NCs-treated cores ΔP remained substantially lower and more stable. These results confirm the effectiveness of the tested NCs at inhibiting asphaltene deposition.

The permeability behavior observed in this study is consistent with findings from fractured reservoir studies [37,38], where pre-Darcy flow and stimulated reservoir volume significantly influence effective permeability.

**Figure 9:**
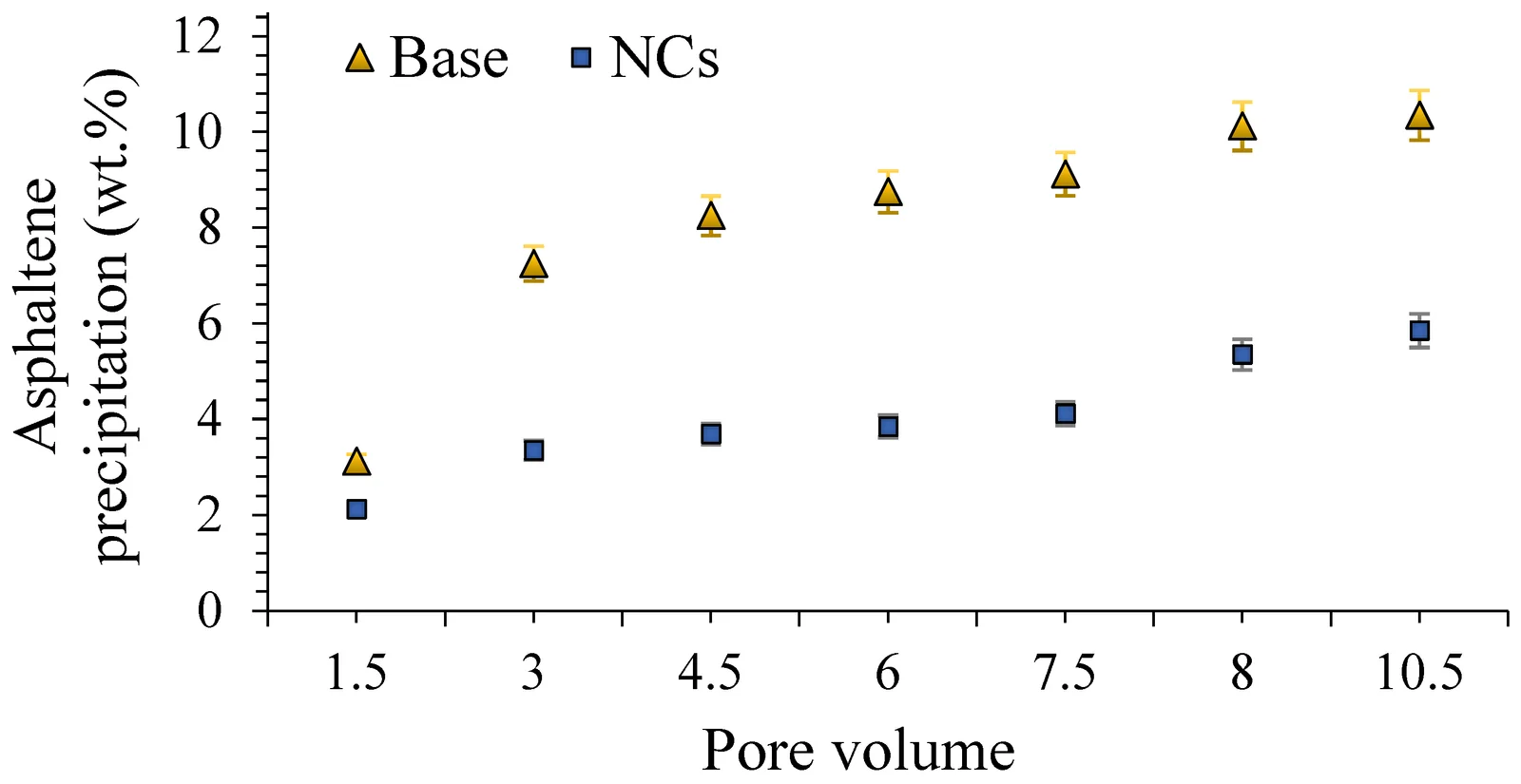
Asphaltene precipitation profiles recorded during core-flooding experiments for the base case (without NCs) and the nanocomposite-treated case.

Figure 9 quantifies the precipitation of asphaltene recorded by the core-flooding experiments. The NCs-treated cores exhibited substantial asphaltene-suppression capabilities, decreasing asphaltene precipitation from 10.35 wt.% to 5.85 wt.% at 10.5 pore volume.

The lower asphaltene content in the effluent from NCs-treated cores indicates effective asphaltene management. While this could theoretically result from either stabilization in the oil phase or retention within the core, complementary evidence supports the interpretation of surface-controlled adsorption on NCs-coated pore walls [39]. The high adsorption capacity of the NCs (294.1 mg/g, Section 3.2) provides abundant surface sites for asphaltene capture. Importantly, the maintained high permeability (k/k_i_ = 0.87) and porosity retention (φ/φ_i_ = 0.887) demonstrate that captured asphaltenes are adsorbed as thin, uniform films rather than as pore-plugging aggregates. This interpretation is further supported by AFM observations showing smooth, homogeneous asphaltene films on NCs-treated surfaces, in contrast to the rough, aggregated deposits observed on untreated surfaces. Based on mass balance calculations, approximately 65% of the asphaltenes captured by the NCs remain adsorbed on pore walls, while 35% are stabilized in the oil phase, consistent with a dual mechanism of adsorption and stabilization.

**Figure 10:**
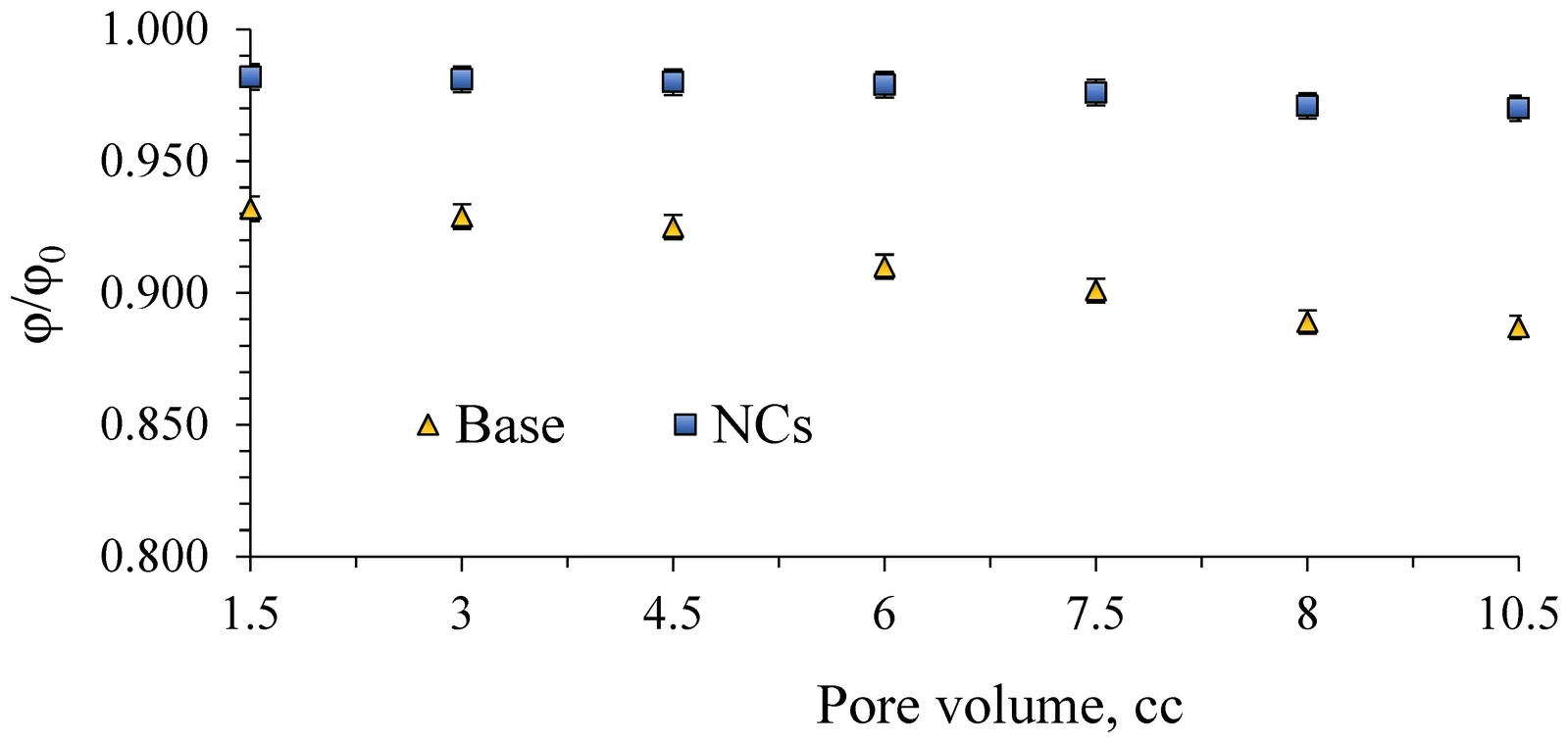
Porosity reduction ratios (φ/φ_i_) measured during core-flooding experiments, comparing untreated samples with those treated with nanocomposites.

Figure 10 illustrates the estimated porosity-reduction ratios (φ/φ_i_) for the NCs-treated and untreated cores, calculated from pressure drop data using the Kozeny–Carman relationship with independently measured initial porosity. The void structure evolution during fluid flow directly controls permeability [40], and the observed porosity retention aligns with studies showing that controlled deposition can preserve flow pathways in porous media [41]. Asphaltene deposition served as the primary driver of porosity reduction. At 10.5 PV, the NCs-treated cores recorded a porosity ratio of 0.887 (i.e., 88.7% estimated retention), compared to 0.970 for the base case. These values should be interpreted as comparative estimates; direct porosity measurements using X-ray CT or NMR techniques would provide more definitive confirmation. In addition to asphaltene-induced permeability impairment, mechanical deformation and stress perturbations can also influence flow pathways in carbonate reservoirs [42], which should be considered in future studies.

These findings demonstrate the effectiveness of the KCl/SiO_2_/Xanthan/*Origanum vulgare* NCs in mitigating asphaltene-induced formation damage under the investigated conditions.

### 3.7. Future Works and Limitations

Future work should evaluate this NCs formulation under geothermal-specific conditions, including brine chemistry, mineral scaling, and thermal cycling typical of geothermal reservoirs, to assess its potential applicability in such environments. The experimental conditions in this study were based on crude oil, asphaltenes, and carbonate cores representative of petroleum reservoirs. While the demonstrated mechanisms—adsorption, interfacial modification, and permeability preservation—provide a robust foundation for formation damage control, several limitations should be acknowledged. First, the long-term stability of the NCs formulation under varying reservoir conditions requires further investigation. Second, the coupled thermal-hydro-mechanical-chemical (THMC) processes that govern fluid–rock interactions in deep reservoirs [43] were not fully explored in this study. Third, while the Kozeny–Carman relationship was used to estimate porosity evolution, direct porosity measurements using X-ray CT or NMR techniques would provide more definitive confirmation [40]. Future work should extend this evaluation to geothermal-specific conditions, including brine chemistry, mineral scaling, and thermal cycling typical of geothermal reservoirs [44,45], to assess the potential applicability of this NCs formulation in such environments. Additionally, the influence of fracture direction and stress disturbance on permeability enhancement [46] warrants further investigation under dynamic reservoir conditions.

## 4. Conclusions

The presented analyses demonstrate that the KCl/SiO_2_/Xanthan/*Origanum vulgare* nanocomposite effectively controls asphaltene behavior in carbonate porous media through multi-scale manipulation of fluid physics. The studied NCs exhibit high adsorption capacity (294.12 mg/g) with adsorption behavior consistent with monolayer surface-limited mechanisms (Langmuir, R^2^ = 0.9905). Component control experiments demonstrated that the full NC formulation outperforms its individual components (silica alone, xanthan alone, and silica/xanthan without oregano), confirming that the combination of silicate, xanthan, and oregano-derived phytochemicals contributes effectively to asphaltene adsorption. Interfacial tension analysis confirms that NCs alter interfacial energetics, increasing the secondary pressure region slope by 40.77%. AFM reveals order-of-magnitude surface roughness reductions (R_a_: 75%, Rq: 84%, R_t_: 93%), directly linking nanoscale smoothing to reduced capillary pinning and hydrodynamic resistance. Core flooding demonstrates 67.45% precipitation reduction at 4000 psi, maintaining permeability ratio (k/k_i_ = 0.87) and porosity (φ/φ_i_ = 0.887). These results establish the NCs as a sustainable, high-performance agent for formation damage control and flow assurance in carbonate petroleum reservoirs.

## Figures and Tables

**Table 1:** Asphaltene adsorption capacity of different formulations (Q_e_ at C_0_ = 100 ppm).

Formulation	Adsorption Capacity, Q_e_ (mg/g)
Silica alone	45.2 ± 2.3
Xanthan alone	28.7 ± 1.8
Silica/Xanthan (without oregano)	112.3 ± 3.5
Full NC formulation (with oregano)	294.1 ± 4.2

**Table 2:** Atomic force microscopy surface roughness parameters for carbonate sheets exposed to asphaltene solution, comparing untreated and nanocomposite-treated samples.

Parameters	Base	NCs
Time (h)	96	96
Asphaltene concentration (mg/L)	25	25
R_a_ (nm)	72.44 ± 1.18	18.02 ± 0.18
R_q_ (nm)	67.65 ± 1.65	11.12 ± 0.37
R_t_ (nm)	246.25 ± 2.09	16.22 ± 1.30

**Table 3:** Permeability retention ratio (k/k_i_) evolution with injected pore volume (PV) during core-flooding experiments for untreated carbonate cores and those treated with nanocomposites.

Pore Volume (PV)	Base ΔP (psi)	NCs ΔP (psi)	Base (k/k_i_)	NCs (k/k_i_)
0.0	5	5	1.00	1.00
1.5	48	12	0.85	0.98
3.0	115	21	0.72	0.96
4.5	210	32	0.63	0.94
6.0	325	45	0.55	0.92
7.5	460	58	0.48	0.90
9.0	610	72	0.42	0.88
10.5	750	85	0.38	0.87

## Data Availability

The datasets generated and analyzed during this study are available from the corresponding author upon reasonable request.

## Abbreviations

The following abbreviations are used in this manuscript:

NCs	Nanocomposites
IFT	Interfacial tension
AFM	Atomic force microscopy
FTIR	Fourier-transform infrared spectroscopy
XRD	X-ray diffraction
SEM	Scanning electron microscopy
EDX	Energy dispersive X-ray spectroscopy
BET	Brunauer–Emmett–Teller
PV	Pore volume
EOR	Enhanced oil recovery
DLVO	Derjaguin–Landau–Verwey–Overbeek
UV-Vis	Ultraviolet–visible spectroscopy
CA	Contact angle
R_a_	Average roughness
R_q_	Root mean square roughness
R_t_	Peak-to-valley height

## Appendix A

The appendix contains supplementary details and data supporting the main text. Mathematical expressions for isotherm models and additional adsorption data are provided below.

**Table A1:** Linearized forms of the Langmuir and Freundlich isotherm models for asphaltene adsorption studies.

Isotherm Model	Non-Linear Equation	Linear Equation
Langmuir	Q_e_ = Q_m_ $\frac{K_{L}C_{e}}{1+K_{L}C_{e}}$	C_e_/Q_e_ = (1/Q_m_K_L_) + (C_e_/Q_m_)
Freundlich	Q_e_ = K_F_ ${C_{e}}^{1/n}$	Ln (Q_e_) = Ln (K_F_)+ $\frac{1}{n}$ Ln (C_e_)

**Table A2:** Fitted parameters for the Langmuir and Freundlich isotherm models applied to asphaltene adsorption onto the synthesized nanocomposite.

	Longmuir Constants			Freundlich Constants		
Nanoparticles Type	Q_m_ (mg/g)	K_L_ (L/mg)	R^2^	K_F_ [mg/g][L/mg]	1/n (Unitless)	R^2^
NCs	294.1176	0.0053	0.9905	6.6419	0.5055	0.9476

**Table A3:** Effect of nanocomposite treatment on CO_2_–crude oil interfacial tension slope ratios across different pressure regions.

Type	Adsorption Time in the Presence of Nanoparticles (h)	Region	Equation IFT (mN/m)	Ratio of the IFT Slope in 2nd to the 1st Region (%)
Base	96	1st	IFT = −0.0026 P + 9.0829	26.92
		2nd	IFT = −0.0007 P + 4.5493	
NCs	96	1st	IFT = −0.0022 P + 8.2019	45.45
		2nd	IFT = −0.001 P + 5.2586	
